# Systemic Infection of Pediatric Osteomyelitis: A Clinical Case in a Low-Income Country

**DOI:** 10.7759/cureus.98326

**Published:** 2025-12-02

**Authors:** Pramodani Arulkumar, Rucha Bhat, Sibana Gonzalez, Aarush R Patlola, Sorivel Sosa

**Affiliations:** 1 Biology, New York University, New York, USA; 2 Public Health, Nova Southeastern University, Davie, USA; 3 Biology, Saint Peter’s University, Jersey City, USA; 4 Chemistry, King Philip Regional High School, Wrentham, USA; 5 Research Department, Global Health Leaders, Arizona, USA

**Keywords:** acute osteomyelitis, clinical case, dominican republic, multidisciplinary care, pediatric, staphylococcus aureus

## Abstract

Osteomyelitis is a severe bone infection that requires early diagnosis and targeted treatment, mainly in pediatric populations where clinical presentations can be complex and nonspecific. This case report presents a seven-year-old male from Los Ríos, Santo Domingo, Dominican Republic, diagnosed with methicillin-resistant Staphylococcus aureus (MRSA)-associated osteomyelitis. The child presented with systemic signs, including fever, vomiting, and abdominal pain, along with localized trauma, limb pain, and functional limitations. Clinical examination revealed hemodynamic instability and gastrointestinal involvement, while laboratory findings indicated infection and malnutrition. Imaging confirmed osteomyelitis in the left femur and periosteal injury in the right forearm. Blood and soft tissue cultures identified MRSA as the causative agent. This case highlights the importance of early imaging, microbial isolation, and a multidisciplinary approach in the management of pediatric osteomyelitis, such as intravenous antibiotics, monitoring, and long-term medications. It also underscores the need for increased awareness and diagnostic capabilities in developing countries to facilitate timely intervention and reduce long-term complications.

## Introduction

Osteomyelitis is the inflammation and infection of bone, characterized by swelling and pain in the affected area. Recent research has advanced understanding of the pathophysiology and potential treatment strategies for osteomyelitis. Osteomyelitis is most commonly caused by pathogens. Hofstee, Muthukrishnan, Atkins et al. (2020) provide a comprehensive review that highlights how Staphylococcus aureus remains the primary causative pathogen in bone infections and describes its ability to penetrate and persist in cortical bone through submicron channels, pores less than one micrometer. They also revisit the critical role of osteocytes in recruiting immune cells to sites of infection and detail how leukocytes, including neutrophils, eosinophils, and basophils, contribute to the diagnosis of implant-associated osteomyelitis [1]. Osteomyelitis can be acute or chronic. Regarding this case study, acute osteomyelitis typically develops over a short period, usually within two weeks after an initial infection or injury, and can present subtly, with nonspecific symptoms like low-grade fever, irritability, or localized limb pain, making diagnosis challenging [2].

For treating osteomyelitis, previous research indicates that antibiotics and, if needed, surgery are the most effective. To remove the diseased bone, surgical debridement can be used, as antibiotics cannot penetrate the abscesses or infected fluids in the body as effectively. Only if surgical debridement cannot be done, then prolonged antibiotic treatment can be extended for months. Regarding the antibiotics themselves, Staphylococcus aureus can be penicillin-resistant or penicillin-sensitive; therefore, alternative antibiotics include clindamycin, vancomycin, or cefazolin [2].

Osteomyelitis is more common in children, particularly those under five, due to several factors related to their developing skeletal and immune systems. Pediatric osteomyelitis most commonly affects the long bones of the arms and legs, especially the femur, tibia, and humerus, but it can affect any bone in the body. These cases are often associated with recent trauma or a minor skin wound that provides an entry point for bacteria. Delayed treatment can lead to complications such as chronic osteomyelitis, growth plate disturbances, or joint dysfunction [2]. This is further facilitated by the presence of transphyseal vessels, blood vessels that cross the growth plate in developing bones, which are more prominent in infants and young children. These vessels provide access for bacteria, increasing the risk of osteomyelitis. The immature immune response in this age group may also hinder the body’s ability to contain and localize infections effectively, leading to rapid disease progression if not promptly diagnosed [3].

Pediatric osteomyelitis requires a tailored approach due to its unique clinical presentation and specific causative pathogens. MRI is considered the gold standard for early diagnosis, enabling detailed assessment of bone involvement. X-ray imaging also differentiates bone infection from neoplastic or non-neoplastic mimickers. Timely diagnosis and targeted microbial isolation are crucial for reducing long-term complications and supporting treatment planning, as well as considering epidemiology [4].

Epidemiological studies have been conducted to determine the proportion of pediatric osteomyelitis cases attributed to each cause. In a study determining the epidemiology of 869 pediatric osteomyelitis cases in the United States, 57.4% were boys and 42.6% were girls, for both chronic and acute cases. Acute cases were more prevalent in younger children, with a median of 9.5 years for acute and 12 years for chronic. 69.1% of cases were caused by Staphylococcus aureus [5]. In a German population younger than 20 years, the highest rate of osteomyelitis was in children ages 10-15 years, and the second highest rate in children ages 5-10 years [6]. In South India, a study analyzed acute osteomyelitis in 207 children with a median age of four years. 69% of cases were male, and S. aureus was the most common gram-positive pathogen [7]. These studies from various countries underscore the significance of early detection and treatment in managing this prevalent disease in children. They also provide a comparison between developed and developing countries, where in developing countries, it is more common to see cases of osteomyelitis in younger children, with the disease being more common in boys than girls [5-7]. Specific statistics on pediatric osteomyelitis cases in the Dominican Republic are largely unknown, as is the location of this case study. Still, it is crucial to emphasize the importance of early detection and treatment in combating this prevalent disease that has impacted children worldwide.

Osteomyelitis presents differently in each affected patient. This case report, therefore, addresses a complex case of osteomyelitis and provides insight into managing similar cases. Examining the background and symptoms of patients will also provide a better understanding of the causes and contributing factors of osteomyelitis, as well as whether there is any correlation between the patient’s background and the severity of their case. A developing country like the Dominican Republic may lack adequate medical facilities in certain areas, so this case report would help those experiencing osteomyelitis symptoms recognize the disease and take appropriate action. The unique features of osteomyelitis in the pediatric context provide a deeper understanding of this disease. Highlighting the course of action and any background events will serve as a guide for doctors treating osteomyelitis in children. This case study reviews and follows an occurrence of pediatric osteomyelitis in a seven-year-old male from Santo Domingo, Dominican Republic.

## Case presentation

A seven-year-old male from Santo Domingo, Dominican Republic, came through the emergency room with symptoms of pain in the right upper limb, trauma to the left lower limb, fever, vomiting, loss of appetite, an absence of bowel movement, and abdominal pain. The order in which symptoms appeared is unknown. His family lived in an urban area with access to good-quality water and adequate waste disposal. The boy weighed 26.4 kg and was 130 cm tall. He had no issues at birth, being the third child of his mother, born at 38 weeks' gestation, and weighing 4.8 kg at birth. In terms of psychomotor development, no issues were noted. He was adequately vaccinated as well.

**Figure 1:**
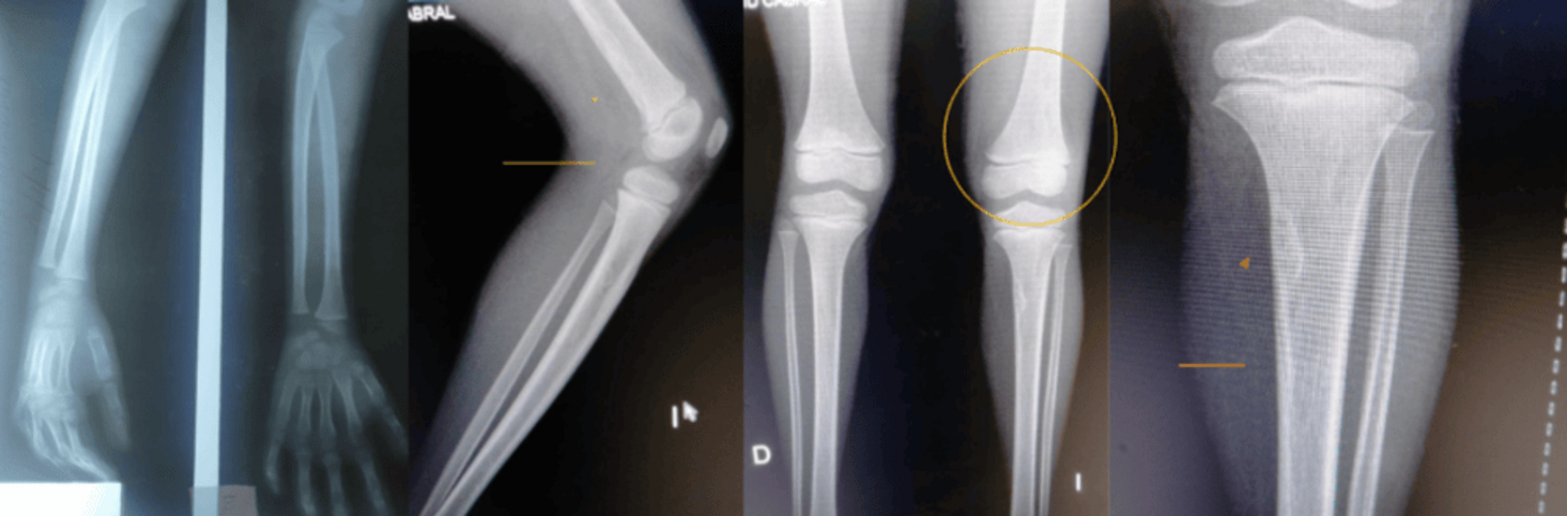
X-ray of right upper limb (left two images) and left lower extremity (right four images)

Upon admission to the emergency department, the seven-year-old male patient presented with multiple alarming symptoms: trauma to the left lower limb, pain in the right upper limb, fever, vomiting, abdominal distension, and absence of bowel movements. On physical examination, the child was alert but febrile (39°C), tachycardic (148 bpm), and visibly distressed. The patient's blood pressure was recorded as 90/60 mmHg. He exhibited pallor of the skin and mucous membranes, weak peripheral pulses, and a capillary refill time exceeding 4s. Signs were consistent with hemodynamic instability. Examination also revealed edema and significant functional limitations in both the right upper and left lower limbs, the latter of which was immobilized in a splint (Figure 1). The abdominal examination showed marked distension, tympanism, and diffuse tenderness, suggesting gastrointestinal involvement.

**Figure 2:**
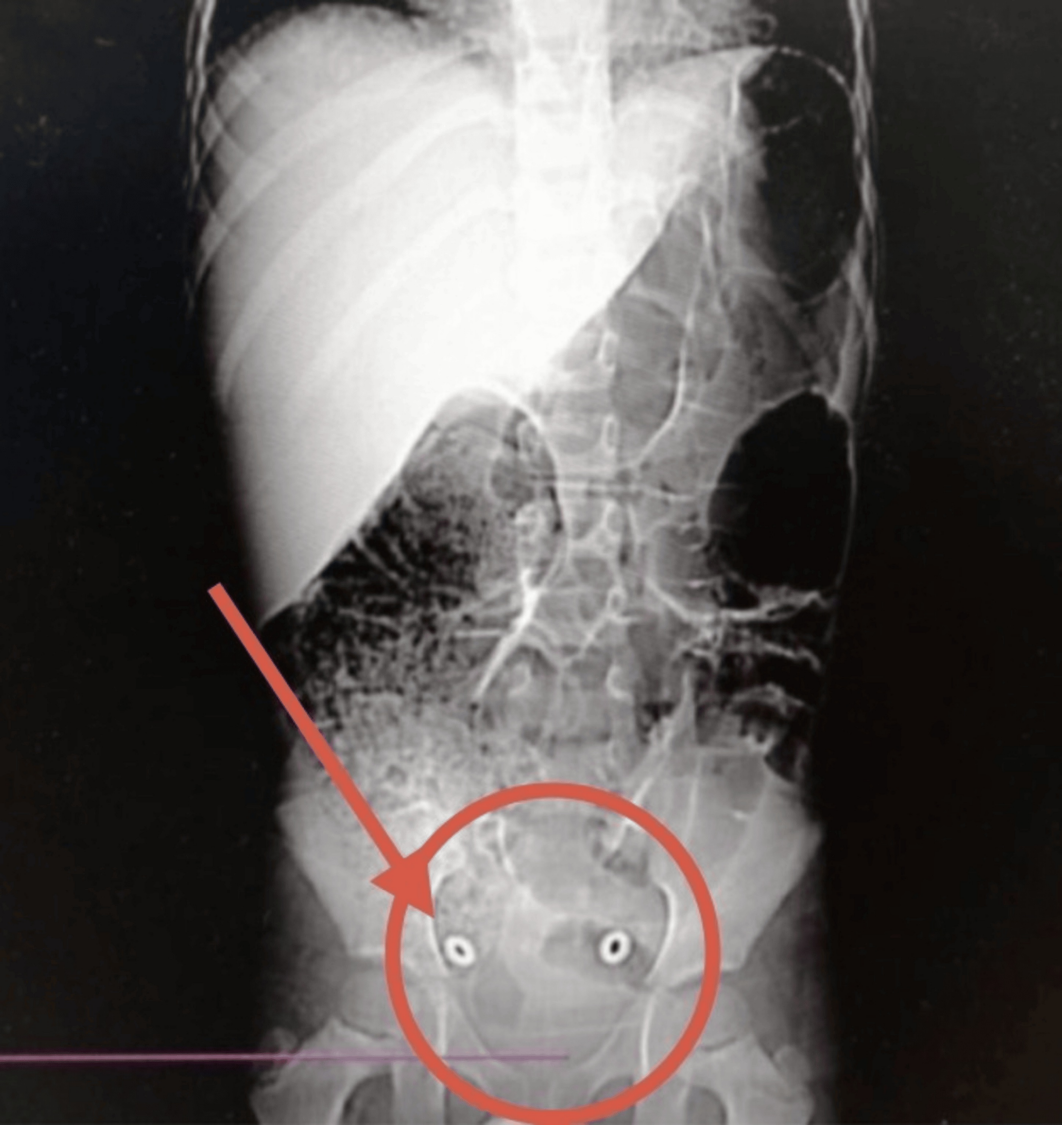
Abdominal X-ray showing fecal impaction, causing the enlarged rectum as indicated by arrow and circle

**Figure 3:**
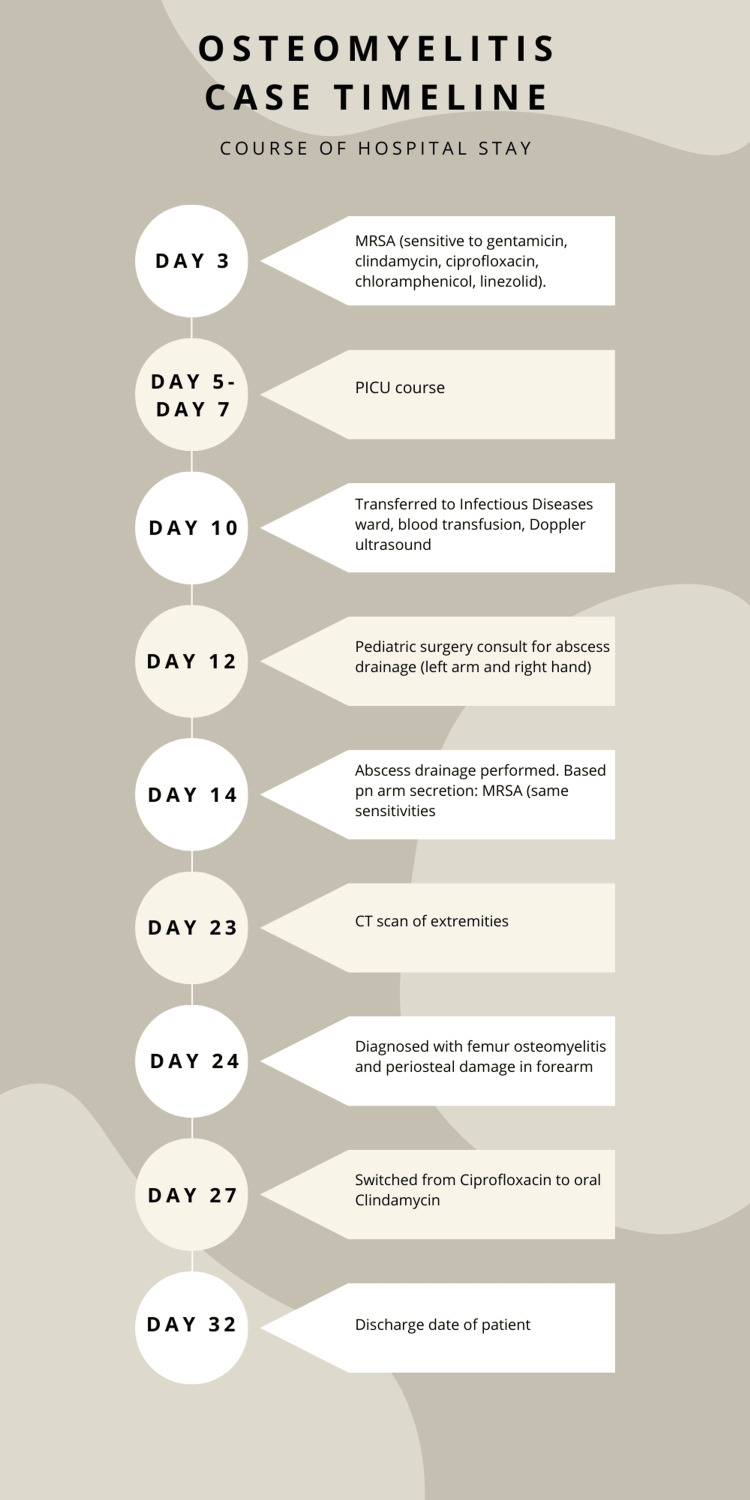
Course of hospital stay from day 3 to day of discharge MRSA: methicillin-resistant Staphylococcus aureus; PICU: Pediatric Intensive Care Unit

Further investigations, including lab work and imaging, revealed several pathological findings: lab results showed anemia (hemoglobin 9.7 g/dL), a high white blood cell count with a predominance of neutrophils, suggesting an infection (Table 1), and low albumin levels (2.4 g/dL, Table 2). Imaging studies, including abdominal X-rays, revealed dilated bowel loops, fecal impaction, and a fecaloma in the right colon (Figure 2). Ultrasound findings included hepatomegaly and biliary sludge. The respiratory assessment revealed significant distress, characterized by intercostal and subcostal retractions, crackles on auscultation, and hypoxemia (PO2 56 mmHg). MRSA was isolated from blood and soft-tissue cultures, confirming staphylococcemia (Table 3). Later imaging and surgical evaluations, such as a bone biopsy, confirmed osteomyelitis of the left femur and periosteal damage in the right forearm. The collection of systemic signs, local musculoskeletal findings, and positive microbiological results confirmed a diagnosis of MRSA-associated osteomyelitis with significant systemic compromise. The hospital stay timeline is shown below (Figure 3).

**Table 1:** Liver function test with results and reference values done on day of admission. AST-ALT: Aspartate transaminase-Alanine transaminase

Liver Function Test	Results	Reference Values
Glucose	158 mg/dL	60-100 mg/dL
AST-ALT	43-33 U/L	0-40 U/L
Creatinine	0.5 mg/dL	0.60-1.30 mg/dL
Urea	16 mg/dL	15-39 mg/dL
Albumin	2.4 g/dL	3.5-5.4 g/dL
Sodium	135.9 mm/L	138-142 mm/L
Potassium	4.0 mm/L	3.5-4.5 mm/L
Chloride	95 mm/L	98-106 mm/L

**Table 2:** Further complete blood count tests done over the course of treatment with reference values [8]

	Day 1	Day 4	Day 5	Day 6	Day 8	Day 10	Day 24	Reference Values
Leukocytes (10^3^)	12.5/mm^3^	11.0/mm^3^	12.0/mm^3^	12.9/mm^3^	14.2/mm^3^	11.5/mm^3^	12.5/mm^3^	4.5-13.5/mm^3^
Lymphocytes	10.1%	15.2%	17.0%	16.9%	14.9%	12.3%	20.3%	20-50%
Granulocytes	82.8%	81.1%	75.8%	77.8%	81.0%	84.3%	76.3%	50-70%
Hemoglobin	9.7 g/dL	7.8 g/dL ↓	8.7 g/dL	7.7 g/dL ↓	9.9 g/dL	8.9 g/dL ↓	9.8 g/dL ↑	11.0-13.5 g/dL
Hematocrit	28.4%	23.4%	26.2%	23.0%	29.0%	26.5%	29.2%	35-45%
Platelets (10^3^)	226/mm^3^	153/mm^3^↓	177/mm^3^	148/mm^3^	194/mm^3^	284/mm^3^	514/mm^3^	160-430/mm^3^

**Table 3:** Microbiology test results over the course of treatment to test for MRSA MRSA: Methicillin-resistant Staphylococcus aureus

Test Done	Day	Results
Blood Culture	3	MRSA Treatment: Gentamicin, Clindamycin, Ciprofloxacin, Chloramphenicol, Lincomycin
Blood Culture	10	No growth
Arm	14	MRSA Treatment: Gentamicin, Clindamycin, Ciprofloxacin, Chloramphenicol, Lincomycin
Blood Culture	25	No growth
Thigh Abscess	26	MRSA Treatment: Gentamicin, Clindamycin, Ciprofloxacin, Chloramphenicol, Lincomycin, Vancomycin

**Figure 4:**
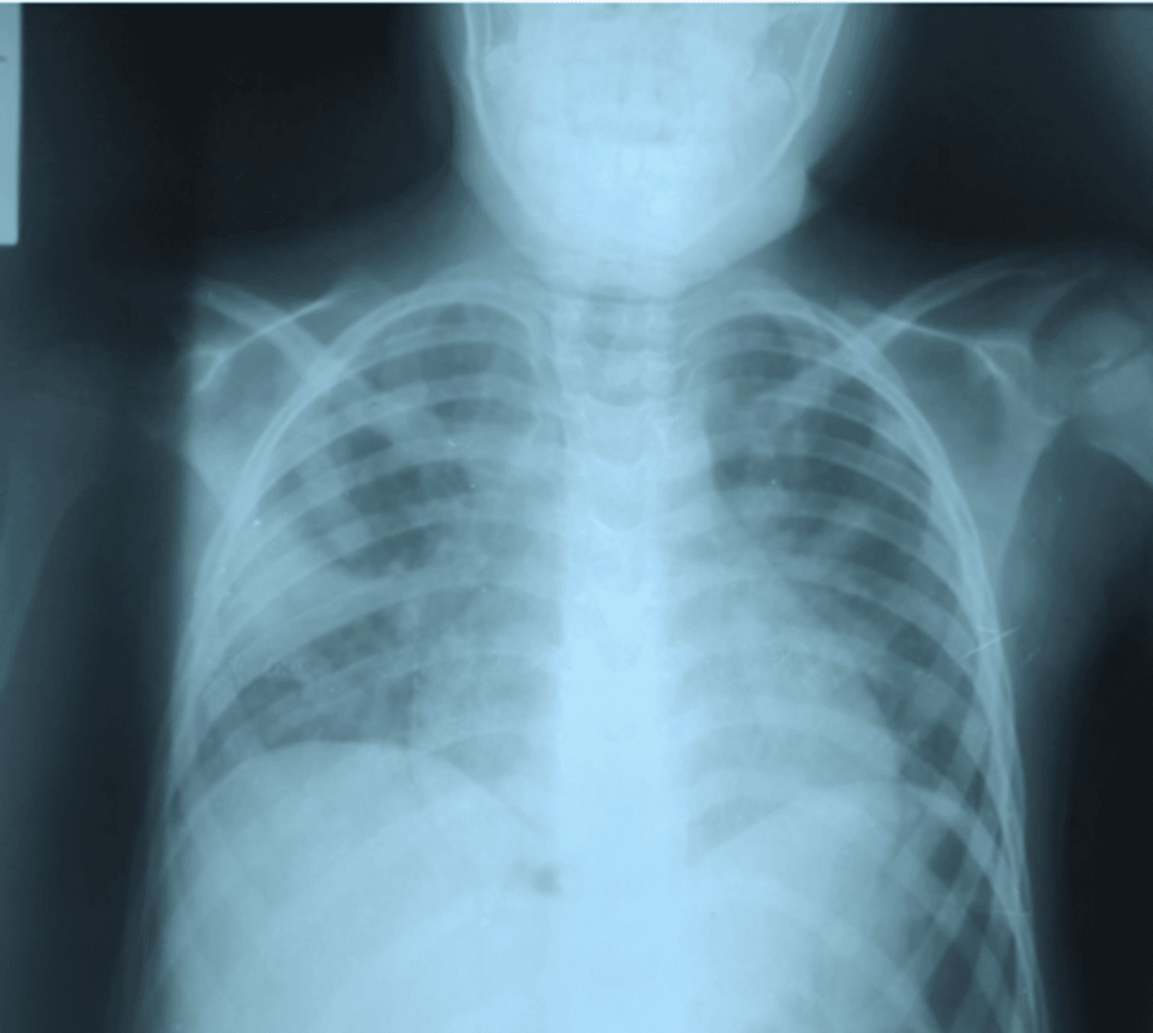
Chest X-ray of patient done on day of admission.

**Figure 5:**
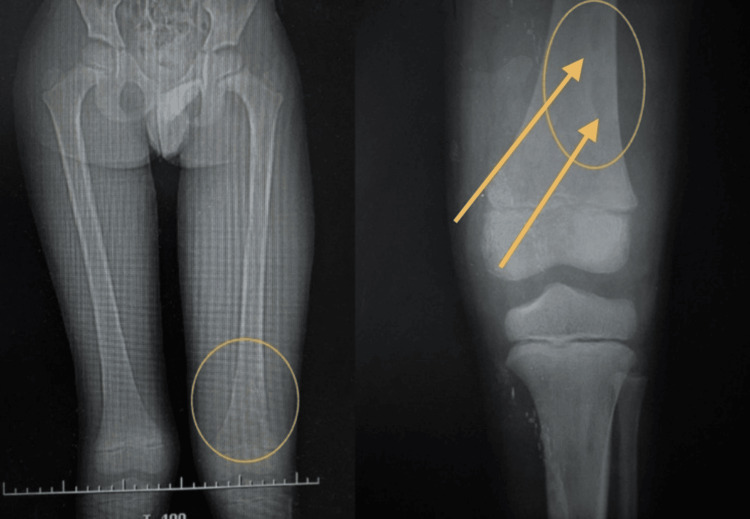
Sonographic images of the left thigh and leg. Circles and arrows display encapsulated abscesses.

**Figure 6:**
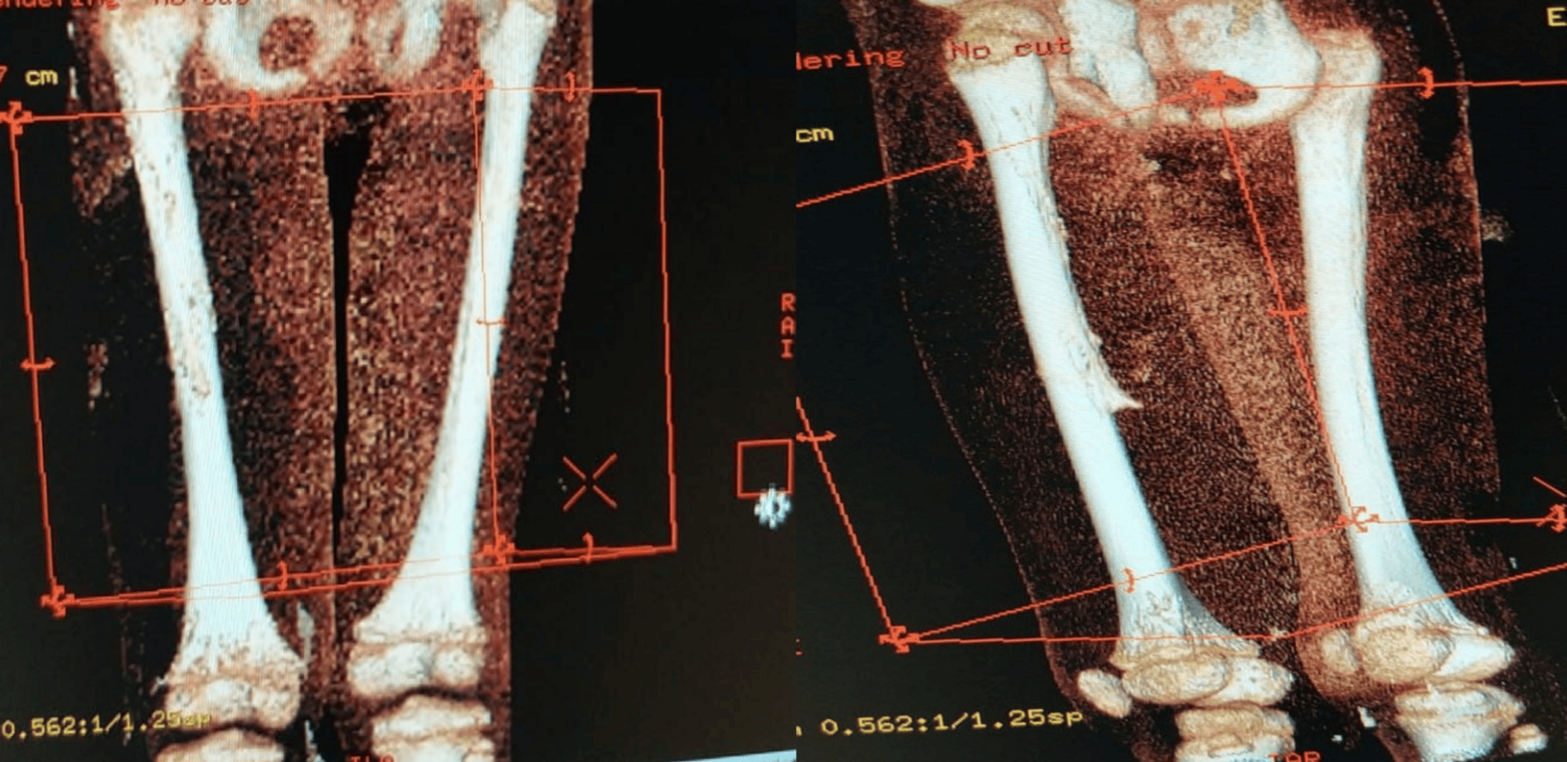
CT scan of lower extremities done on day 23.

X-rays of the patient’s chest (Figure 4) were also performed to visualize the disease course. Initial diagnoses included probable staphylococcemia, trauma to limbs, and fecal impaction. Sonographic images of the left leg displayed encapsulated abscesses (Figure 5). Finally, a CT scan of the lower extremities was performed on day 23 to confirm the extent of disease (Figure 6). Combined with imaging conducted during the hospital stay (previously mentioned), a final diagnosis of femur osteomyelitis and periosteal damage in the forearm was reached on day 24.

The therapeutic management of the 7-year-old male patient with multifocal MRSA osteomyelitis, soft tissue abscesses, sepsis, and fecal impaction was multifaceted, involving pharmacologic, surgical, and supportive interventions over a 31-day hospital stay. Initial treatment began in the emergency department with empiric intravenous antibiotics like ceftriaxone and vancomycin, which provided broad coverage against both Gram-positive and Gram-negative organisms. However, with worsening clinical signs and confirmation of MRSA on blood cultures, the antibiotic regimen was escalated to piperacillin-tazobactam and linezolid, offering broader-spectrum activity and improved soft-tissue penetration, especially against MRSA. Once the patient stabilized, ciprofloxacin was introduced and continued for 17 days due to its excellent bone penetration, and eventually replaced by oral clindamycin. These changes were guided by microbiological results and clinical progress, with later cultures confirming the absence of ongoing infection.

Surgical intervention was also necessary to control localized infections. On day 14, the patient underwent abscess drainage procedures on the left arm and right hand following a pediatric surgery consultation. Imaging studies, including a CT scan of the extremities, confirmed the diagnosis of femur osteomyelitis and periosteal damage in the forearm. Despite these findings, there is no mention of bone debridement or more invasive orthopedic surgeries, suggesting a successful response to antimicrobial therapy and drainage alone.

Secondary care played a critical role throughout the patient’s hospitalization. He received intravenous fluids according to the Holliday-Segar method to maintain hydration, and 100% hypertonic fluid resuscitation was administered in the Pediatric Intensive Care Unit (PICU) in response to hemodynamic instability, marked by pallor, prolonged capillary refill, and weak peripheral pulses. Due to low serum albumin levels, human albumin was administered to restore oncotic balance. Anemia, evidenced by a hemoglobin level of 9.7 g/dL, was treated with multiple packed red blood cell transfusions. Pain and fever were managed with analgesics and antipyretics as needed.

During his PICU stay, the patient developed respiratory distress requiring mechanical ventilation. Sedation was maintained with midazolam and fentanyl, and oxygen therapy was initially provided via a binasal cannula. Blood gas analysis indicated respiratory compromise, warranting ventilatory support. Nutritionally, enteral feeding was initiated via a nasogastric tube, and a Foley catheter was used to monitor urinary output.

Gastrointestinal symptoms, including vomiting, abdominal distension, and fecal impaction, were managed with polyethylene glycol as a laxative and omeprazole to protect the gastric lining, under the guidance of a gastroenterologist. Abdominal imaging revealed dilated bowel loops and a fecaloma, and physical examination supported a diagnosis of impaction without mechanical obstruction.

Throughout treatment, multiple specialty services were involved, including infectious diseases, gastroenterology, pediatric surgery, and orthopedics. The patient showed gradual improvement, with serial cultures turning negative and his condition stabilizing. He was discharged after 31 days of hospitalization, with a final diagnosis of osteomyelitis and MRSA-associated sepsis. His treatment underscores the importance of early, aggressive intervention, multidisciplinary care, and pathogen-directed therapy in managing complex pediatric infections.

Follow-up treatment was determined by the hospital's orthopedic department upon reevaluation on day 26. It was confirmed there was osteomyelitis of the left femur, and continued physical therapy, and medications were given to progress treatment. Examples of physical therapy included postural training, passive exercises, and respiratory therapy. The patient was also given ambulatory management upon discharge. Ambulatory management consisted of multiple antibiotic medications. Ceftriaxone and vancomycin were prescribed for four days, piperacillin/tazobactam and linezolid were prescribed for seven days, ciprofloxacin for seventeen days, and clindamycin for five days. After oral administration of the medication, the patient's treatment was complete.

Throughout the initial treatment period, the patient received intravenous antibiotics. Therefore, there were no gaps in adherence to the treatment plan as medical professionals controlled all medication administration. Treatment was then switched to oral clindamycin as the infection stabilized. The patient did not have worsening symptoms during this time, suggesting that the treatment regimen was effective and that there were no significant gaps in care. No adverse events related to the medical treatment occurred throughout the hospital stay. The unexpected symptomatic respiratory distress, which proved to be respiratory compromise, was quickly managed with mechanical ventilation. The patient did not have any recorded unexpected medical incidents during the period after the hospital stay through any follow-ups.

## Discussion

This case of multifocal MRSA osteomyelitis in a seven-year-old male underscores the significant diagnostic and therapeutic challenges associated with pediatric bone infections, particularly within global contexts where access to specialized pediatric imaging and infectious disease expertise may be limited. Osteomyelitis in children often begins with vague, nonspecific symptoms like fever, irritability, gastrointestinal upset, or limb pain that can easily be attributed to benign illnesses or unrelated conditions [4]. In this case, the presence of trauma and fecal impaction further complicated the clinical picture, illustrating how overlapping symptoms can obscure the early recognition of osteomyelitis. This diagnostic delay is not unusual; early osteomyelitis can mimic soft-tissue injuries, septic arthritis, transient synovitis, or occult fractures, and distinguishing between these conditions is difficult without appropriate imaging and laboratory evaluation.

The child’s rapid progression of symptoms aligns with known pediatric physiology. Children are predisposed to hematogenous osteomyelitis due to the unique structure of their metaphyseal blood supply, which allows bacteria to lodge and proliferate in areas where sluggish capillary flow meets nutrient-rich marrow [1,9]. As supported by epidemiological research, Staphylococcus aureus remains the most common causative pathogen worldwide, especially community-associated MRSA strains, which are increasingly reported in pediatric populations [7,9]. This case reinforces these trends, as MRSA was confirmed as the source of infection.

The diagnostic process described incorporates MRI, CT imaging, and culture, which align with current best-practice recommendations. MRI is considered the gold standard because it can detect marrow edema, abscesses, periosteal elevation, and soft-tissue extension well before radiographs become abnormal [2,3]. The successful use of multimodal imaging in this case aligns with evidence emphasizing early imaging to prevent complications such as chronic infection, growth plate destruction, or systemic sepsis [5,10,11]. Research consistently shows that delays in imaging and diagnosis significantly worsen outcomes in children, especially in multifocal disease, where symptoms may be widely distributed [6].

The management of this case, including both surgical intervention and prolonged, targeted antibiotic therapy, is consistent with the literature on optimal treatment strategies for pediatric osteomyelitis. Surgical debridement remains essential when abscesses, necrotic bone, or periosteal collections are present, and MRSA infections in particular often require aggressive early intervention due to their virulence and propensity for rapid tissue destruction [1,12,13]. Clinical outcome studies emphasize that the combination of timely debridement and pathogen-directed antibiotics greatly improves prognosis, reduces hospitalization duration, and lowers long-term complication rates [5]. This multidisciplinary approach is critical in limited-resource settings where delays in access to surgery or IV antibiotics can exacerbate disease severity.

Another significant element in this case is its global health context. While pediatric osteomyelitis is well-described in North America and Europe, there is far less published data from regions such as the Dominican Republic. The relative lack of regional research means that clinicians may have limited information regarding local pathogen prevalence, antimicrobial resistance patterns, or typical clinical presentations [7]. This data gap is concerning, as epidemiological studies show that osteomyelitis incidence is influenced by socioeconomic conditions, access to trauma care, nutritional status, and regional variations in bacterial strains [6]. The scarcity of research from the Caribbean makes this case particularly important, as it contributes to a growing global understanding of how pediatric osteomyelitis presents and progresses in diverse settings.

Overall, this case strongly supports the existing literature, which emphasizes early recognition, advanced imaging, and integrated surgical-medical management as cornerstones of effective osteomyelitis treatment. It also highlights the ongoing need for improved surveillance, reporting, and clinical resources in understudied regions. By contributing to broader epidemiological knowledge, cases like this help promote earlier recognition and better outcomes for children globally.

## Conclusions

The case reinforces the value of a multidisciplinary, dynamic treatment strategy in pediatric osteomyelitis, particularly when complicated by MRSA. Empirical antibiotic therapy was appropriately escalated following positive cultures and worsening symptoms, illustrating the critical need for pathogen-directed therapy in managing resistant organisms. Surgical drainage, without the need for more invasive orthopedic intervention, further demonstrates how timely, targeted intervention can limit long-term damage. Supportive care, like fluid management, transfusions, respiratory support, and nutritional therapy, was just as vital in stabilizing the patient during his PICU stay. The collaborative efforts of infectious disease specialists, gastroenterologists, surgeons, and intensivists contributed to the patient’s recovery. This case not only emphasizes the importance of early diagnosis and intervention but also highlights how structured clinical protocols and interdepartmental coordination can lead to favorable outcomes, even in severe, multifactorial cases of pediatric osteomyelitis.

## References

[1] Hofstee MI, Muthukrishnan G, Atkins GJ (2020). Current concepts of osteomyelitis: From pathologic mechanisms to advanced research methods. Am J Pathol.

[2] Patel D (2025). Osteomyelitis: Practice essentials, anatomy, pathophysiology. Medscape.

[3] Restrepo R, Park HJ, Karakas SP (2024). Bacterial osteomyelitis in pediatric patients: A comprehensive review. Skeletal Radiol.

[4] Momodu II, Savaliya V (2023). Osteomyelitis. https://www.ncbi.nlm.nih.gov/books/NBK532250/.

[5] Disch K, Hill DA, Snow H, Dehority W (2023). Clinical outcomes of pediatric osteomyelitis. BMC Pediatr.

[6] Walter N, Bärtl S, Alt V, Rupp M (2021). The epidemiology of osteomyelitis in children. Children (Basel).

[7] Jaybhaye A, Lg S, Dash N (2023). Clinical spectrum and microbial etiology of bone and joint infections in children: A retrospective analysis from South India. Am J Trop Med Hyg.

[8] American Academy of Pediatrics (2016). Pediatric care. AAP Pediatric Care Online.

[9] Saavedra-Lozano J, Falup-Pecurariu O, Faust SN (2017). Bone and joint infections. Pediatr Infect Dis J.

[10] World Health Organization. (2020 (2025). GLASS report: Early implementation 2020. https://www.who.int/publications/i/item/9789240005587.

[11] Berbari EF, Kanj SS, Kowalski TJ (2015). 2015 Infectious Diseases Society of America (IDSA) clinical practice guidelines for the diagnosis and treatment of Native vertebral osteomyelitis in adults. Clin Infect Dis.

[12] Urish KL, Cassat JE (2020). Staphylococcus aureus osteomyelitis: Bone, bugs, and surgery. Infect Immun.

[13] Peltola H, Pääkkönen M (2014). Acute osteomyelitis in children. N Engl J Med.

